# Effectiveness and safety of surgical treatment of carpal tunnel syndrome via a mini-transverse incision and a bush hook versus a mid-palmar small longitudinal incision

**DOI:** 10.1186/s13018-022-02967-z

**Published:** 2022-02-05

**Authors:** Dongyue Wang, Tianxiao Ma, Yuqing Hu, Xiaocui Zhao, Lihua Song

**Affiliations:** Department of Orthopaedic Surgery, The General Hospital of Jizhong Energy Xingtai Mining Group, No. 202 Bayi Street, Xingtai, Hebei People’s Republic of China

**Keywords:** Bush hook, Mini-transverse incision, Proximal wrist crease, Carpal tunnel syndrome, Small longitudinal incision, Effectiveness and safety

## Abstract

**Background:**

Minimally invasive surgery for carpal tunnel syndrome has been consistently the mainstay of treatment. In this study, we developed a novel bush hook via a mini-transverse incision at proximal wrist crease to surgically treat carpal tunnel syndrome and our aim was to compare the results with those of mid-palmar small longitudinal incision in carpal tunnel release.

**Methods:**

This is a retrospective study on patients who received a mini-transverse incision and a novel bush hook or a mid-palmar small longitudinal incision for treatment of carpal tunnel syndrome. The decision to receive either technique was made mainly based on patients' choice. The clinical results were evaluated at 1 week, 1 month, 3 and 6 months postoperatively and compared.

**Results:**

In total, 58 patients in mini-transverse incision group and 74 in mid-palmar longitudinal incision group were include. The follow-up period was 6.8 ± 1.6 months. The mini-transverse incision group had a significantly smaller incision (4.3 ± 0.4 mm vs. 26.2 ± 1.6 mm), shorter surgical time (7.8 ± 2.6 min vs. 19.7 ± 2.8 min), but not for hospital stay (3.2 ± 1.9 vs. 3.6 ± 2.2 days). Both groups showed significant improvement from baseline level at any time points postoperatively (all *P* < 0.001). At 1 month and 3 months, the mini-transverse incision group showed a significantly better improvement of VAS, SSS and FSS score (*P* < 0.05). At 6 months, the differences were no longer significant (*P* > 0.05). In addition, the mini-transverse incision group showed a significantly reduced time to return to the work and activities, tendency to higher rate of excellence and good outcomes and fewer complications.

**Conclusions:**

This novel technique via a mini-transverse incision and bush hook showed better clinical effectiveness and safety, and can be considered as an alternative for wrist tunnel release after the results are validated by higher-level evidence studies.

*Evidence level*: III.

**Supplementary Information:**

The online version contains supplementary material available at 10.1186/s13018-022-02967-z.

## Introduction

Carpal tunnel syndrome (CTS) is a leading cause for neuropathy in the upper limbs, with a prevalence of 3.8% in the general population [1], and higher in manual technicians or wrist joint high-frequency users [2, 3]. Surgery is a preferable choice of treatment, particularly in those not cured via repeated conservative treatments. Compared to traditional open surgery for which effectiveness was compromised by established flaws of unshapely hypertrophic scars and symptomatic pain, minimally invasive technique via limited or mini-incision has gained more popularity, due to marked advantages concerning operative trauma, appearance and scar-related complications [4–7].

Numerous attempts for minimally invasive surgery have been introduced into practice for treatment of CTS, including endoscopic carpal tunnel release (ECTR) and various kinds of small incision surgery (mid- or root-palmar longitudinal incision, wrist palmar transverse incision) [6, 8]. These methods each had their own advantages or disadvantages; however, no one method is absolutely superior to the other one [9]. Furthermore, most of them were small-sample pilot study, and the reported clinical results have hardly been validated. In our practice, we developed a novel mini-profile bush hook via a 3–5 mm mini-transverse incision at the proximal wrist crease to achieve the purpose of median nerve decompression. This technique is expected to have the theoretic advantages and potential to obtain better improvement in symptoms and wrist functions due to the minimal incision than any previously reported and less trauma to the soft-tissue. By far, their clinical effectiveness and safety have not been investigated, especially in contrast to other carpal tunnel release (CTR) techniques.

In this study, our aim was to compare the clinical results and postoperative complications of a mini-transverse incision approach via a novel bush hook verses mid-palmar small longitudinal incision in carpal tunnel release.

## Methods

### Study design

This was a retrospective study. The local ethnic committee approved the study and all the participating patients provided the written consent before the study commencement.

Patients who presented with CTS and received minimally invasive surgery via either a minimal transverse incision in 1 length of 3–5 mm and a novel bush hook or a mid-palmar longitudinal incision of 3 cm in length for CTR were included in this study for assessment. CTS was diagnosed and confirmed by clinical symptoms (nocturnal pain or paresthesia, numbness in the median nerve distribution), physical examination (Tinel or Phalen signs, or significantly reduced muscle strength to grasp object between two fingers) and positive electromyography findings. Patients who had persist symptomatic pain but were unresponsive to conservative management (rest, bracing, nonsteroidal anti-inflammatory (NSAID), injection, physiotherapy or others) for at least 6 months were considered as surgical candidates. The exclusion criteria included age of < 18 or > 70 years old, history of any surgery at the affected wrist or hand, presenting with bilateral CTS to undergo bilateral surgical procedures.

The decision to receive either technique was made mainly based on the patients' choice after they were informed of the potential benefits and postoperative complications of each procedure; for patients who had no preference for either procedure, the surgical decision was left to their attending surgeon' discretion.

### Surgical techniques

#### Mini-incision and novel bush hook

**Fig. 1:**
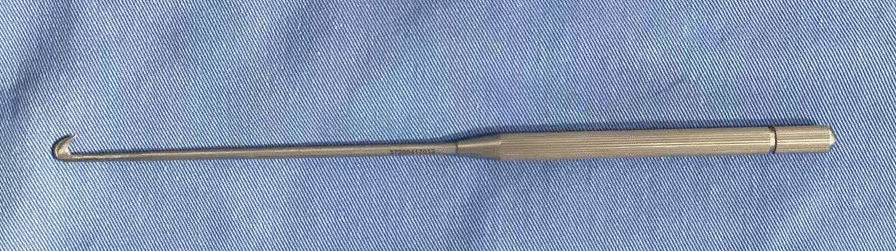
Profile of the mini-bush hook, 4.7 mm in height and 0.8 mm in width, featuring the two skids (the tip was blunt) with the sandwiched sickle-shaped blade

The bush hook is a knife made of stainless steel entirely, for specific use in CTR surgery. It measures of 4.7 mm in height and 0.8 mm in width, featuring the two skids (the tip was blunt for purpose of protecting the tissues from cutting trauma) with the sandwiched sickle-shaped blade between for division of the flexor retinaculum (Fig. 1).

**Fig. 2:**
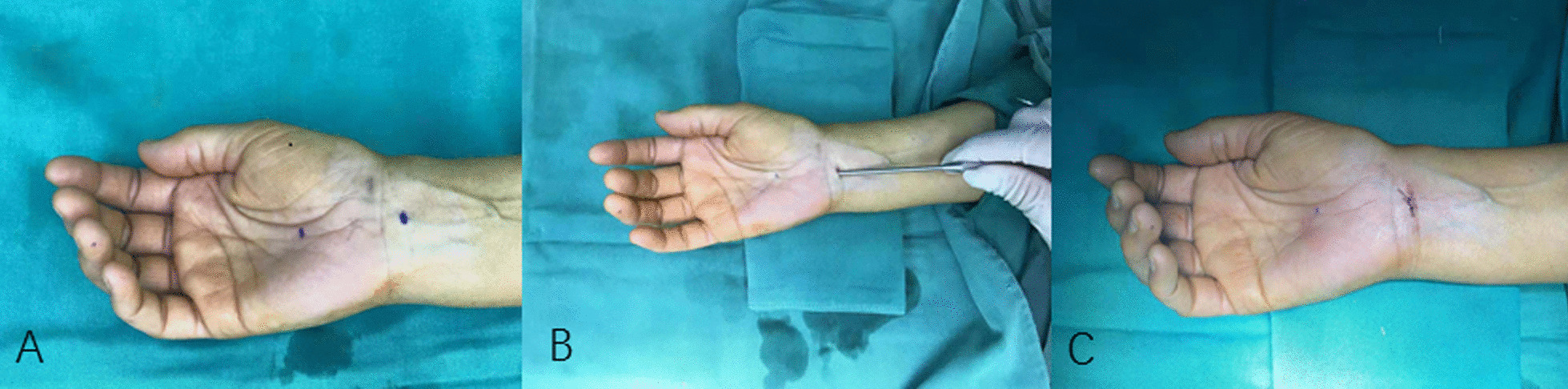
**a** The starting point and ending point were marked. **b** The mini-bush hook was introduced into the wrist tunnel up to the ending point and pulled back to divide the flexor retinaculum. **c** The typical mini-incision about 4–5 mm at the proximal wrist crease and closed by one suture

Under local anesthesia, the forearm was abducted and a padding was placed beneath the dorsal wrist joint to keep the wrist extend at 20°–25°. A transverse mini-incision in length of 3–5 mm was made, with the midpoint located at the intersection of the proximal transverse wrist crease and the 3rd webspace line (3WL). Eye or iridectomy scissors was used to dissect the skin and soft tissues to expose the superficial layer of the aponeurosis of the flexor superficial digitorum tendon, preserving the aponeurosis. The small-profile bush hook was introduced into the wrist tunnel and advanced distally along the 3WL, with its tip upwards and keeping paranal to the long axis of the forearm, until to the intersection of Kaplan's cardinal line (KCL) and 3WL [10]. At this point, the sudden “give” will be felt, indicating the tip just distal to flexor retinaculum. Then, the bush hook knife was slowly drawn back proximally to cut off the transverse carpal ligament; if necessary, repeated cuttings can be performed for complete release (Additional file 1 and 2). Careful hemostasis and surgical site cleaning were done, and 2/0 or 3/0 nylon suture was used to close the skin. The procedure is depicted in Fig. 2.

#### Limited incision at the mid-palm

**Fig. 3:**
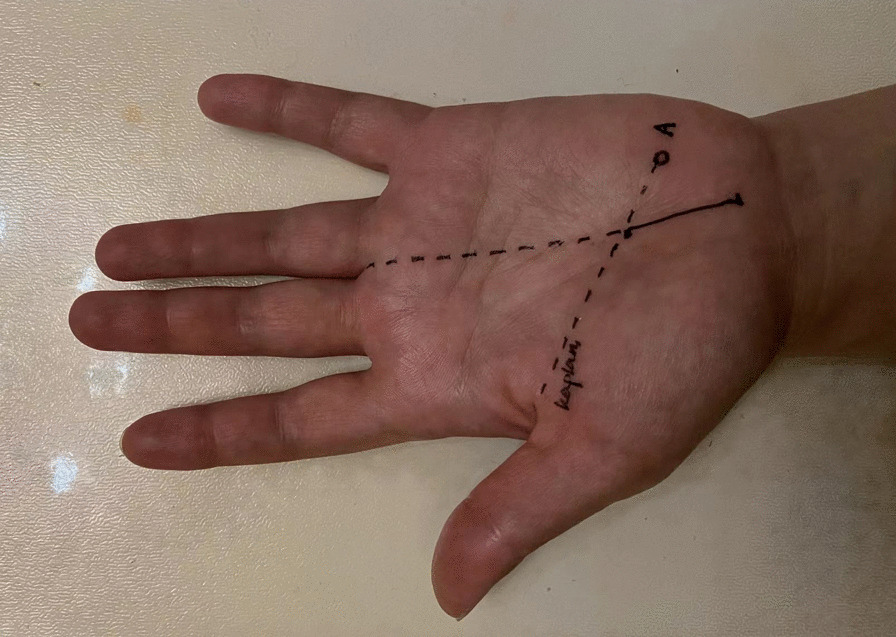
The location of the small longitudinal incision at the mid-palm was marked

Brachial plexus block anesthesia or local anesthesia was applied. The forearm was abducted, with a padding beneath dorsal wrist joint to keep them extend at 20°–25°. A longitudinal incision of 2.5–3.0 cm in length was made starting at the thenar crease at the base of the palm and marked and ending at the intersection point of the extended 3rd web line (3WL) and Kaplan' cardinal line (Fig. 3). The skin and subcutaneous tissue were incised and elevated with a retractor to expose the transverse carpal ligament and median nerve. Then, transverse carpal ligament was incised longitudinally with scissors at the ulnar side of the median nerve, along the 3WL. Median nerve entrapment trace could be clearly observed, and if necessary, epineurial release was performed. Careful hemostasis and surgical site cleaning were done, and 2/0 or 3/0 suture was used to close the skin.

#### Outcome measures

Perioperative parameters were recorded, including incision length (mm), surgical time (minutes) and hospital stay. Hospital stay was determined by inquiring the medical records, and the time (days) from surgery to return to previous work and activities was recorded at postoperative follow-ups.

Visual analogue scale (VAS) and Boston carpal tunnel questionnaire (BCTQ) were measured at preoperation, 1 month, 3 months and 6 months, postoperatively. The BCTQ was a self-administered scoring system by patients and consisted of 11 items for symptom severity subscale (SSS) and 8 items for functional severity subscale (FSS), with a possible score of 1–5 for each item; the final scoring points (range 1–5) for each subscale were calculated by the sum of scores for items divided by the number of items. Total score ranges from 1 to 5 [11]. The overall outcome at the last visit was evaluated using Kelly's proposed grading scale [6], which was developed according to relief of symptoms for CTR and widely used in subsequent studies [12, 13]. This scale graded the surgical outcome to be: excellent (complete relief of symptoms), good (persistence of occasional minor symptoms), fair (some constant or annoying symptoms) and poor (symptoms unchanged or worse).

Intraoperative or postoperative complications at each visit were recorded, including potential surgical site infection, hematoma, scar area pain or pillar pain, injuries to the recurrent branch or palmar cutaneous branch of median nerve, injury to superficial palmar arch, injury to the tendons or hypertrophic scars.

### Statistical analyses

Continuous data were presented with mean and standard deviation (SD), and the normality was evaluated by the Shapiro–Wilk test. Within-group comparisons were performed using paired sampled *t*-test to investigate the improvements from baseline (preoperative) to postoperative measurements at each visit. Between-group comparisons were performed using independent sampled Student *t* test or Mann–Whitney *U* test, as appropriate. Categorical data were presented with number and percentage, and were compared using *X*^2^ test. A two-sided alpha level of 0.05 or less was considered significant. All the analyses were performed using SPSS 24.0 (IBM Corporation, Armonk, New York).

## Results

Initially, 144 patients were enrolled to receive small longitudinal incision surgery (*n* = 65) or the mini-transverse incision surgery with use of the novel bush hook (*n* = 79). Seven participants in the former group and 5 in the latter group were lost to follow-up, thus leaving 58 and 74 in either group for data analyses.

Both groups exhibit similar characteristics in demographics (age, 49.6 ± 11.8 years vs. 47.3 ± 13.5 years; female predominance, 79.3% vs. 77.0%), affected side (right, 62.1% vs. 56.8%), affected limb dominance (65.5% vs. 58.4%), duration of symptoms (18.6 ± 6.2 vs. 15.9 ± 7.3 months) or any comorbidity (all *P* values > 0.05) (Table 1). Either nonsignificant difference was observed in VAS (3.9 ± 1.3 vs. 3.9 ± 1.7, *P* = 0.972), SSS (3.2 ± 0.7 vs. 3.3 ± 0.7, *P* = 0.692) or FSS score (3.3 ± 0.7 vs. 3.4 ± 0.8, *P* = 0.824). The mean follow-up period was 6.6 ± 1.2 and 6.9 ± 1.7 months in either group, not significantly different (*P* = 0.827) (Table 2).

**Table 1:** Comparisons of demographics, medical conditions and comorbidities between mini-transverse incision group and small longitudinal incision group

	Mini-transverse incision group (*n* = 58)	Small longitudinal incision group (*n* = 74)	*P*
Age (year)	49.6 ± 11.8	47.3 ± 13.5	0.793
Sex			0.753
Male	12 (20.7)	17 (23.0)	
Female	46 (79.3)	57 (77.0)	
Affected side			0.538
Right	36 (62.1)	42 (56.8)	
Left	22 (37.9)	32 (43.2)	
Dominance			0.403
Dominant	38 (65.5)	45 (58.4)	
Non-dominant	20 (34.5)	29 (41.6)	
Duration of symptoms	18.6 ± 6.2	15.9 ± 7.3	0.218
Comorbidities			
Hypertension	17 (29.3)	18 (24.3)	0.520
Diabetes mellitus	9 (15.5)	9 (12.2)	0.577
Ischemic heart disease	6 (10.3)	10 (13.5)	0.580
Hyperlipidemia	14 (24.1)	17 (23.0)	0.875
Hyperuricemia	6 (10.3)	8 (10.8)	0.931
Follow-up period (months)	6.6 ± 1.2	6.9 ± 1.7	0.827

Data presentation: mean ± standard deviation (SD) or number (percentage)

**Table 2:** Comparisons of surgery-related parameters and clinical results between mini-transverse incision group and small longitudinal incision group

	Mini-transverse incision group (*n* = 58)	Small longitudinal incision group (*n* = 74)	*P*
Incision length (mm)	4.3 ± 0.4	26.2 ± 1.6	< 0.001
Surgical time (minutes)	7.8 ± 2.6	19.7 ± 2.8	0.001
Hospital stay (days)	3.2 ± 1.9	3.6 ± 2.2	0.692
Days to return to work	9.2 ± 3.5	20.8 ± 3.9	0.001
VAS			
Preoperation	3.9 ± 1.3	3.9 ± 1.7	0.972
Postoperative 1 month	1.1 ± 0.7	1.8 ± 0.9	< 0.001
Postoperative 3 months	0.4 ± 0.6	0.9 ± 0.6	0.007
Postoperative 6 months	0.2 ± 0.4	0.5 ± 0.5	0.363
SSS assessment			
Preoperation	3.2 ± 0.7	3.3 ± 0.7	0.692
Postoperative 1 month	2.1 ± 0.7	2.6 ± 0.6	0.003
Postoperative 3 months	1.8 ± 0.5	2.0 ± 0.6	0.214
Postoperative 6 months	1.3 ± 0.5	1.5 ± 0.5	0.475
FSS assessment			
Preoperation	3.3 ± 0.7	3.4 ± 0.8	0.824
Postoperative 1 month	2.2 ± 0.7	2.5 ± 0.6	0.023
Postoperative 3 months	1.9 ± 0.6	2.2 ± 0.7	0.092
Postoperative 6 months	1.3 ± 0.6	1.4 ± 0.7	0.675
Kelly' grades			0.820
Excellent	42 (72.4)	45 (65.2)	
Good	11 (19.0)	15 (21.7)	
Fair	4 (6.9)	7 (10.1)	
Poor	1 (1.6)	2 (2.9)	

Data presentation: mean ± standard deviation (SD) or number (percentage); *VAS* visual analogue scale, *SSS* symptom severity subscale, *FSS* functional severity subscale

The transverse incision group had a significantly smaller incision in length (4.3 ± 0.6 mm vs. 26.2 ± 1.6 mm, *P* < 0.001) and shorter surgical time (7.8 ± 2.2 vs. 19.7 ± 2.8 min, *P* = 0.001) than those did the longitudinal incision group. Hospital stay was not significantly different between groups (3.2 ± 1.9 vs. 3.6 ± 2.2, *P* = 0.692) (Table 2).

Both groups showed significantly improvements from baseline level (preoperation) for any time points postoperatively, and all the within-group comparisons showed the significant results (all *P* < 0.001).

At postoperative 1 month and 3 months, the mini-transverse incision group showed a significantly better improvement for outcome measurements, except for a nonsignificant but tendency toward a better FSS at postoperative 3 months (*P* = 0.092). At postoperative 6 months, both groups showed the nonsignificant result, for either VAS, SSS or FSS improvement (*P* = 0.363, 0.475, 0.675, respectively).

The time to return to the previous work and activities was significantly shorter in mini-transverse incision group (mean, 9.2 ± 3.5) than in longitudinal incision group (mean, 20.8 ± 3.9). The rate of excellence and good for transverse incision was 91.4% (excellence, 72.4%; good, 19.0%) and for the longitudinal incision was) 87.0% (excellence, 65.2%; good, 21.7%).

In the longitudinal incision group, scar area pain or pillar pain was found in 2 cases, injury to the recurrent branch of median nerve in 1 case and hypertrophic scar in 1 case. In the transverse incision group, no complications were observed.

## Discussion

Currently, mini-incision technique using a variety of mini-incisions or novel instruments for surgical treatment of CTS has gained increasing popularity. In this study, we used a novel mini-profile bush hook via a transverse incision of 3–5 mm in length at proximal wrist crease to achieve the purpose of the complete CTR. Compared to the commonly used small longitudinal incision technique, this mini-transverse incision technique showed significantly advantages in operative trauma, early functional recovery, tendency toward fewer complications and allowing more quickly return to work and activities.

There were several newer approaches or instruments advocated for CTR surgery. Faraj et al. [14] compared the effectiveness of mini-transverse incision at distal wrist crease without endoscope-assisted versus the traditional open technique and found the higher rate of satisfaction by symptomatic relief (90% verse 80%), better cosmetic appearance (scar length, 1.4 cm vs. 5.2 cm) and faster return to the daily activities. With shape somewhat similar as our mini-bush hook, KnifeLight has been extensively used during the last two dozen. The biggest advantage of KnifeLight is the light source installed at the distal “skids,” which allows it possible to locate precisely the tool blade by transillumination; namely, the procedure is completed under partial visualization [15]. Despite with such advantage, the relatively large incision than ours (12-25 mm vs. 4.3 mm) and no standard protocol formed as to the incision locations (variably used) and incision lengths may partially compromise getting the consistent clinical results [4, 16, 17]. Li et al. [18] used the mini-hook knife combined with dilating metal catheter for CTR in their preliminary 12 cases of CTS and reported more safety that avoided the injury to nerves, but the limited number of cases should be considered an issue that affected its extrapolation. In this study, we have got the similar favorable results as those in previous literature, but only use of an incision of about 1/8–1/4 in length as that and more importantly hidden within the proximal wrist crease, demonstrating this technique' effectiveness and the theoretic merit in reducing any scar-related complications.

Our minimal invasive technique using mini-bush hook can be an alternative to the endoscopic technique for CTR. As an early minimally invasive technique, endoscopic CTR has advantage in significantly reducing surgical site-related complications, but is limited to widespread use due to high cost of equipment and setup and need for a steep learning curve, increased risk of iatrogenic injury to nerve or blood vessel and occasionally the incomplete release [6, 9, 13]. In contrast, our minimally invasive technique is promising in widespread use for second- or primary-level medical institutions where endoscopic equipment was not affordable or not readily available, due to the very low cost, simple and easy operation to perform, hardly need of specific learning and more importantly the similar clinical results.

As with other limited incision techniques, identification of anatomical features especially the boundaries of the “safe area” and their relationship with flexor retinaculum and neighboring neurovascular structures is key to avoiding surgery-related complications [7, 19, 20], this study used the proximal wrist crease, flexor retinaculum and 3WL as the topographic landmarks to keep the procedure safe and effective. It is of note, despite with variations, the motor recurrent branch of the median nerve is generally described 5 mm or more distally to the 3rd web space, which makes the 3WL a safe zone for operation even in “blind” operation [19]. During operation, the cutting is sure to keep in consistently straight with 3WL for division of flexor retinaculum, thus avoiding the injury to the cutaneous palmar branches or ulnar nerves, since it has the lowest innervation density of the base of the palm [21]. Our finding that no cases of injury to the nerves were found in the mini-transverse incision group confirmed the feasibility and necessity for use of these landmarks, in particularly for those completely “blind” procedures.

In the longitudinal incision group, we found 2 cases of scar area pain or pillar pain. The reason may be that the small longitudinal incision cut off the pillar skin and subcutaneous tissue bridge, resulting in mechanical injury [7, 14]. Moreover, this longitudinal incision is prone to scar adhesion and inducing scar pain, in contrast to that the mini-transverse incision is located hidden within the proximal transverse wrist and about 1/7 in length which thus hardly at risk of scar pain or pillar pain. In addition, injury to the recurrent branch of median nerve was observed in one patient in the longitudinal incision group, which may be attributable to the incomplete exposure of the recurrent branch of median nerve through longitudinal wrist incision.

This study had several limitations. First, patients were not randomized and the decision to either treatment regimen was primarily made by the patients themselves. This may have compromised the comparison results, because patients would have their own inclination to either technique before they were admitted and possibly reported the biased results. The nonsignificant differences between two groups for the demographics or CTS conditions might partly compensate or counteract for this bias. Second, the follow-up period was relatively short as 6 months, although patients in both groups obtained satisfactory symptomatic and functional improvement within this period. Third, this was a pilot study and thus did not include so many participants. Nevertheless, the results have showed remarkable merits over one commonly used technique. Therefore, the difference will only be more significant in statistics, if larger sample is supposed. Fourth, this is a single-center study, and the better design studies will be required to validate our findings.

In conclusion, we introduced this novel mini-profile bush hook via a mini-transverse incision at the proximal wrist crease for CTR and demonstrated the better early clinical effectiveness and safety over the small longitudinal incision technique. This technique can be considered as an alternative technique after the results are validated by higher-level evidence studies.

## Supplementary Information

**Additional file 1:** The detailed surgical procedure using the novel bush hook for CTR via the a mini transverse incision (First).

**Additional file 2:** The detailed surgical procedure using the novel bush hook for CTR via the a mini transverse incision (Second).

## Data Availability

All the data will be available upon motivated request to the corresponding author of the present paper.

## Abbreviations

CTS: Carpal tunnel syndrome
ECTR: Endoscopic carpal tunnel release
CTR: Carpal tunnel release
NSAID: Nonsteroidal anti-inflammatory
3WL: 3rd webspace line
KCL: Kaplan's cardinal line
VAS: Visual analogue scale
BCTQ: Boston carpal tunnel questionnaire
SSS: Symptom severity subscale
FSS: Functional severity subscale
SD: Standard deviation
